# The Spiral of Attention, Arousal, and Release: A Comparative Phenomenology of Jhāna Meditation and Speaking in Tongues

**DOI:** 10.1002/ajhb.24189

**Published:** 2024-12-09

**Authors:** Josh Brahinsky, Jonas Mago, Mark Miller, Shaila Catherine, Michael Lifshitz

**Affiliations:** ^1^ Division of Social and Transcultural Psychiatry McGill University Montréal Quebec Canada; ^2^ Integrated Program in Neuroscience McGill University Montréal Quebec Canada; ^3^ Monash Centre for Consciousness and Contemplative Studies Monash University Melbourne, Victoria, Australia; ^4^ Department of Psychology University of Toronto Toronto Ontario Canada; ^5^ Meditation Teacher San Jose California USA; ^6^ Lady Davis Institute for Medical Research, Jewish General Hospital Montréal Quebec Canada

**Keywords:** high arousal contemplation, Jhāna meditation, speaking in tongues

## Abstract

Buddhist Jhāna meditation and the Christian practice of speaking in tongues appear wildly distinct. These spiritual techniques differ in their ethical, theological, and historical frames and seem, from the outside, to produce markedly different states of consciousness—one a state of utter calm and the other of high emotional arousal. Yet, our phenomenological interviews with experienced practitioners in the USA found significant points of convergence. Practitioners in both traditions describe a dynamic relationship between focused attention, aroused joy, and a sense of letting go or release that they describe as crucial to their practice. This paper highlights these shared phenomenological features and theorizes possible underlying mechanisms. Analyzing our phenomenological data through the lens of various theories of brain function, including sensory gating and predictive processing, we propose that these practices both engage an autonomic field built through a spiral between attention, arousal, and release (AAR).

## 1 Introduction

Jhāna meditation and the practice of speaking in tongues seem wildly different. Jhāna practitioners produce a quiet, seated focus, an outward appearance of utter calm and inner stillness. Their practice follows the intricate threads of a Buddhist tradition, meticulously outlined in texts that precisely guide focused attention to a meditation object such as the breath. During Jhāna, practitioners aim to become fully absorbed in a sequence of meditative states characterized by stability, equanimity and/or bliss. In stark contrast, those engaged in speaking in tongues utter what seem like nonsensical sounds, their expressions marked by loud cries, wails, laughter, and even dance. Speaking in tongues is a Christian charismatic evangelical practice of releasing control of the voice and allowing the inspiration of the Holy Spirit to guide prayer. This tradition draws inspiration from brief passages in the New Testament, but has largely been passed down orally with historical roots in the Black Church (Hollenweger 1988). The practice lacks detailed written documentation, and participants often describe the experience as arising spontaneously. There is no mistaking one for the other, at least on the surface.

While practitioners of Jhāna and people who speak in tongues rarely share philosophical views or social attributes, we were surprised that our phenomenological interviews often indicated points of convergence. In this paper, we will show these unexpected commonalities between seemingly disparate practices, and in the process, highlight overlapping experiential features that may point toward shared underlying mechanisms. Our exploration reveals that participants in both practices oscillate between focused attention, aroused joy or bliss, and the experience of release or letting go. This may be because both practices aspire toward a state of deep mental involvement where a person's attention is fully immersed in the perception of an activity, thought, or experience, often to the exclusion of extraneous stimuli—what psychologists call “absorption” (Tellegen and Atkinson 1974). In that pursuit, attention, arousal and release, (what we call AAR), emerge as co‐creative forces, mutually supportive mechanisms across diverse spiritual spaces.

“Religious” emotions are often conceptualized by researchers as distinct from the mundane ebbs and flows of everyday feeling. In Christianity, a seemingly awkward mix between abject sadness and utter joy, or the simultaneous coexistence of terror and bliss, is often described by observers and practitioners (i.e. Otto 1958). Buddhist meditators, on the other hand, may strive for a highly disciplined form of equanimity, unperturbed by the emotional tapestry of our everyday lives (Ñ. Bhikkhu 1995, 123–25, 503–5). At the furthest extremes of these emotional fields lie, the practice of Jhāna, characterized by deep quietude, and tongues prayer, which emanates a rare intensity of visible arousal. However, we propose that beneath their apparent disparities, these two practices might share a family of experiential, cognitive, and perhaps neurobiological patterns.

Jhāna practitioners aim to develop undistracted attention by focusing on a chosen meditation object, such as the breath. They report that as concentration develops, engagement with sensory perceptions, personal narratives, and discursive thoughts gradually ceases. At the same time, they describe that feelings of energy and joy arise. These conditions of high arousal can be followed by a striking experience of letting go that seems to usher the mind toward a sense of immersion.

And in reverse order, speaking in tongues emphasizes letting go of control as an act of submission to God, which often results in flamboyant and highly aroused emotional experiences. The precursor is an intense focus of submission to God—a passionate focus, albeit a focus nonetheless. And then, at the zenith of fervent worship, people often recount an experience of serene and quiet peacefulness. In other words, although deeply different, both Jhāna and speaking in tongues involve an oscillating process of focus and joy, arousal and release.

Put schematically (see Table 1.), the AAR framework suggests that as we focus on an object, it becomes clearer, boosting the brain's confidence in its perceptual model of the object, thereby associating the object with positive feelings. These positive feelings make attention less effortful, creating a reciprocal feedback cycle between attention and arousal. Throughout, practitioners consciously intend to surrender individual thoughts, self‐oriented desires, and personal control, a process made easier by the emerging sense of effortlessness fostered by this cycle. As the spiral of attention, arousal and release progresses, it leads to deeper states of absorption.

**TABLE 1:** Comparing Jhāna Meditation and Speaking in Tongues.

	Jhāna	Tongues
Attention	Calm, quiet focus on the breath *nimitta* leads to states of absorption called Jhānas.	Passionate loving attention on God is held throughout the practice.
Arousal	*Pīti*, or joy, emerges in the preparation for the early Jhānas, can be quite intense, but is expected to dissipate into equanimity as practice deepens.	Aroused emotions often arise throughout the practice, with catharsis an important lens for experiencing God, but also considered a potential distraction.
Release	Practitioners release everyday thoughts to focus on the breath *nimitta* and then surrender all effortful control of their attention as they enter absorption.	Practitioners release everyday thoughts, and the muscles of their tongues and speech to God, and in moments of especially intense connection will release full bodily, emotional, and cognitive control.

In this paper, through extensive phenomenological data, we explore the relationship between attention, arousal and release across Jhāna meditation and tongues prayer. We draw inspiration from Garland and Frederickson, who propose that mindfulness practice is characterized by an interconnected upward spiral between decentering, broadened attention, and reappraisal (Garland et al. 2010, 2015). Here we propose another kind of spiral—the AAR spiral—as a framework to understand the reciprocal influence between attention, affect and effortlessness in contemplative practice. Our model also leverages emerging predictive processing theories of brain function, which understand the brain as a system that is constantly making predictions about the world and updating those predictions based on incoming sensory data. Analyzing our phenomenological data through this lens, we attempt to explain the emergence of an autonomic field built in the spiraling between AAR, through which sympathetic and parasympathetic systems converge in a choreography of mutual coactivation.

## 2 A Comparison

At a recent conference we posted a few quotes from interviews we conducted with practitioners of Jhāna and speaking in tongues, followed by a brief description of the two practices. We asked the audience if the descriptions seemed more like Jhāna or more like speaking in tongues. For example, consider the following quote. From which tradition does it seem to come?

I let go of everything…It feels like you're falling. The first few times that happened to me, it was terrifying. I like to call it “slipping upward” because it feels like a lifting to me…

How about this next one?

It feels like a constant invitation to let go of more control, and the more control I let go the more powerful an experience it is. I'm shaking. I'm trembling. I feel sensations in my body which I believe is having given up more control. And the more I let go, the lighter I feel. The more set free from all the cares in the world and the worries I have on my shoulders, it makes me want to laugh at myself…All of a sudden, I have that perspective that I'm just like a little teeny dot on the earth [amid] galaxies upon galaxies…

Can you tell which is speaking in tongues and which is Jhāna? Some people did get these right, but our audience was pretty much evenly split.

When read in full, the cultural framing becomes easily visible.

I just set this intention. “May I enter the first Jhāna? A happiness born of seclusion,” that's my mind trigger. And then I just let go of everything. I let go of the intention, I let go of everything. And the mind goes into Jhāna from there. It feels like you're falling. The first few times that happened to me, it was terrifying. I like to call it “slipping upward” because it feels like a lifting to me, upward into Jhāna.

And for speaking in tongues:

It feels like a constant invitation to let go of more control, and the more control I let go the more powerful an experience it is. I'm shaking. I'm trembling. I feel sensations in my body which I believe is having given up more control. And the more I let go, the lighter I feel. The more set free from all the cares in the world and the worries I have on my shoulders, it makes me want to laugh at myself. They seem so menial and small when you're in touch with how great the Lord is. All of a sudden, I have that perspective that I'm just like a little teeny dot on the earth and he's made galaxies upon galaxies. All of a sudden I'm in touch with how in control he is.

We can do a very similar exercise with arousal, although we will leave it to you to read it with and without the underlined sections:

It feels like your body becomes a power plant. Like that same subtle joy that I explained to you…magnify that by like 1000. And that's what it feels like in Jhāna for me. That's pīti, that's the thing that arises. It feels like I could power a whole city, is what it feels like….it feels like my bones are breaking, they are shattered by intense joy.

Then compare that to this one:

I could feel almost a vapor on my hands. Think of steam coming out of your hands. There is just a gentle feeling when you can sense the Holy Spirit. The other way that I know that God is present, is that nothing matters at that point. Oh, actually this is how I would describe it, when you are in a steam room and you can feel the sweat beads running down your cheek and you don't want to move. You're so hot, you're enduring. That is how it feels. It's like you don't want to even move because his presence is just running over. Oh, it's hot…You just want to let the process happen. I am paying close attention, I am focused on what is happening with me and the spirit, or just adoring God. I think it's your heart is filled, your perspective is changed, your outlook, your stance. You get filled with this inner strength and peace, whatever it is.

The point here is simple. Across these two very different practices, practitioner reports of the experience can sound quite similar. So similar, in fact, that we wonder if the underlying mechanisms might overlap.

## 3 Literature Review

In the realm of contemplative science, the predominant focus has gravitated toward serene practices within Buddhist traditions. However, as anthropologist Erika Bourguignon points out, nearly all cultures also include high arousal contemplative practices (Bourguignon 1973). Whitehouse and Lanman augment this conversation by proposing a dichotomy between imagistic rituals, often imbued with heightened arousal, and doctrinal rituals, which tend toward a calming disposition (Whitehouse and Lanman 2014). Likewise, scholars have distinguished between drunk and sober practices in Sufism, with drunkenness referring to the absence of wits during a powerful influx of ecstasy (Mojaddedi 2003). Hasidics are known for their exuberant celebrations (Leshem 2014), while West African and Caribbean possession rituals also involve intense emotions (Brown 2001). Many psychedelic experiences (Preller and Vollenweider 2018) also elicit high arousal states. The practitioners we interviewed for this study blend both arousal and serenity, touching on the far edges of the contemplative landscape.

In the world of neuroscience, while most work has been on serene contemplation, some attention has been paid to high arousal practices. Hagerty and colleagues collected functional magnetic resonance imaging (fMRI) data from individuals in self‐described Jhāna states, showing that their dopamine and opioid reward systems can be hyperactivated through volitional mental activity, without the need for external cues or rewards (Hagerty et al. 2013). Similarly, Kozhevnikov and colleagues, through a series of studies, uncovered sympathetic activation patterns across various Vajrayana meditation techniques, which they argued enhance cognitive function by increasing phasic alertness (Kozhevnikov et al. 2022). They also found that g‐tummo meditation elevates peripheral and core body temperatures (Amihai and Kozhevnikov 2014, 2015; Kozhevnikov et al. 2009). And relevant research into charismatic evangelicals speaking in tongues suggests a dampening of frontal lobe activity (Newberg et al. 2006).

What our participants describe as “release” has a close corollary in recent research suggesting that meditative training typically begins with significant effort but, over time, becomes more effortless (Davidson and Lutz 2007). Neuroscientific studies have shown that advanced meditation is associated with increased activity in sensory processing areas and reduced activity in regions involved in cognitive control (Tang, Hölzel, and Posner 2015). Some researchers have argued that the feeling of ease in contemplative practices can arise from a reorientation of values and conceptual frameworks, such that over time we think more positively of the practice and so feel more motivated to engage (Brewer, Davis, and Goldstein 2013). Here, we suggest that practices like Jhāna and speaking in tongues may also involve a sense of release at a more basic perceptual level, drawing our attention effortlessly to the object of contemplation.

Existing research in psychology lays the groundwork for investigating a dynamic and reciprocal relationship between attention, arousal and release in contemplative practice. The Yerkes–Dodson's effect suggests an optimal level of arousal for task performance; too little or too much arousal can impede cognitive functioning (Yerkes and Dodson 1908). This implies that arousal significantly shapes attentional focus, suggesting a delicate balance that individuals maintain through attentional mechanisms.

Furthermore, Csikszentmihalyi's concept of “flow” (Csikszentmihalyi 1990) may also reflect an interplay between attention and arousal. Flow represents a state of consciousness where individuals are fully immersed in an activity, often leading to improved performance, a sense of release and profound satisfaction. Achieving flow requires a delicate equilibrium between challenge and skill, akin to the interplay between attention and arousal. The proposed spiral relationship between AAR could manifest during flow experiences as attention is directed toward the task at hand, arousal increases to match the challenge, and a subsequent sense of release is experienced upon successful engagement. However, it is noteworthy that in Jhāna meditation and speaking in tongues, a sense of release appears to be interwoven throughout the process, rather than just coming at the end.

During Jhāna, people in our study focus on the perception of the breath. At high levels of concentration, a *nimitta* can emerge, a perception of an internally generated mental object which most often appears as a light or a diffuse sense of luminosity. Lindahl and colleagues suggest that this perception of a light‐like *nimitta* could be the result of a homeostatic mechanism that upregulates sensory systems when they are gated by intense focus (Lindahl et al. 2014). We also build from connections between attention and arousal that have been established through a predictive processing framework (Joffily and Coricelli 2013; Seth and Friston 2016; Hesp et al. 2021).

## 4 Subjects and Methods

Our neurophenomenological method (Varela 1996; Timmermann et al. 2023) aims to establish connections between the phenomenology observed in speaking in tongues and Jhāna meditation and recent advances in the science of attention, positive emotions, and predictive processing. Employing a “front‐loading” approach to neurobiological data collection, we integrate phenomenological interviews and extensive participant observation, spanning several years in the case of tongues prayer (Shaun Gallagher 2003; S Gallagher 2012; Lifshitz, Sheiner, and Kirmayer 2018; Lutz and Thompson 2003; Cardeña and Terhune 2014). This comprehensive preliminary phase laid the groundwork for subsequent brain scanning protocols, incorporating functional magnetic resonance imaging (fMRI), electroencephalography (EEG), and autonomic measures. Through these methods, we aim to systematically map the diverse experiential dimensions that unfold during the practice of contemplation (Brahinsky, Lifshitz, and Luhrmann 2024). This paper is built upon the phenomenological elements of our research.

Phenomenology of this sort relies on the idea that first person accounts not only provide meaningful descriptions of inner experience (Petitmengin and Bitbol 2009; Petitmengin and Lachaux 2013), but also that the words and concepts we use shape those experiences. In essence, the spiral between attention, arousal and release is mediated by both language and practice. Lakoff, Johnson and colleagues provide insight into this relationship by contending that metaphor is not merely a linguistic construct but an integral facet of human thought intricately linked to embodied experiences (Lakoff and Johnson 2003). They suggest that metaphorical mappings can influence not only language, but also perception, action, and even physiological responses. Our project advances upon these findings to explore the neurophenomenology of what appears to be a relationship between attention, arousal and release that is mediated by metaphor and practice.

The Christian phenomenological data for this study draws from interviews with US based charismatic evangelical Christians in the Mind and Spirit Project (*n* = 40), a multimodal initiative funded by the Templeton Foundation and led by Tanya Luhrmann, as well as a neurophenomenological study on prayer (*n* = 66) funded by the US National Science Foundation and the Bial Foundation, led by Tanya Luhrmann, Josh Brahinsky, and Michael Lifshitz.

Interviews with Buddhist Jhāna practitioners (*n* = 10) were gathered as part of the collaborative initiative “Cognitive Mechanisms of Spiritual Practice” involving the Berkeley Social Interaction Lab led by Dacher Keltner and the Laboratory of Michael Lifshitz at McGill University, led by Jonas Mago with support from Dharma College Berkeley and the Monash Centre for Consciousness and Contemplative Studies. The Jhāna practitioners attended a 10‐day meditation retreat in Georgia, USA, taught by Shaila Catherine, an American Buddhist teacher and co‐author on this paper. For this study, the interviews occurred in the weeks following the conclusion of the retreat.

## 5 Attention During Jhāna Practice

Jhāna practice is an advanced concentration meditation in the Buddhist tradition during which focus becomes so intense that many aspects of ordinary conscious experience fade from view. But, while the outward appearance is of deep calm, the inner experience, particularly of early phases of practice as the mind prepares for Jhāna, can be highly energized and suffused with rapturous joy. The participants in our study used the breath or *nimitta* (a sign of concentration most commonly perceived as a mental experience of light) as their meditation object. They described that the focus on the breath and the internal light of the *nimitta* seem to generate increased joy as well as deep calm. In a feedback loop, the joy and calm then appear to support the consistent focus on the breath *nimitta*. As attention settles on the breath *nimitta*, meditators become increasingly insensitive to ordinary sensory experience (Laukkonen et al. 2023).

One participant, Arnold, for instance, describes how Jhāna manifests in utter stillness. “When I'm in a deep, concentrated state, but I'm not in an absorption [Jhāna], there are quick, subtle movements of thoughts, and images. They're going so fast. But when I go into a jhānic state, that movement disappears. And it's a stillness.” This stillness manifests after sustained focus on the breath or *nimitta* and produces a sense of internal steadiness. As Shane put it, “When attention is given to that kind of mental object [referring to the *nimitta*], it strengthens it. Whereas when attention is given to bodily sensations or changing mental factors, they break apart; they are perceived as arising and passing.” Instead, the *nimitta*, “becomes a suitable object for the steadiness of mind.”

## 6 Release During Jhāna Practice

Jhāna practitioners use the word release in a variety of contexts. It may refer to a cognitive practice of letting thoughts, obstructive habits, or desires dissipate by recognizing that they are based on imaginal constructs. Release can also describe a way that practitioners actively and intentionally give up the sense of being the agent who is applying attention. Giving up control of their mind–body processes and personal construct of self seems to allow relaxation and trust to carry them deeper into meditative experience. In this manner, Jhāna practitioners recognize the refinement of attention as both the precursor and the effect of letting thoughts fall away. They embrace release as a metaphor that shapes practice, and points toward the aspirational goal of their path. In one sense of the word, release might seem opposed to the rigor implied by practices that focus attention, and yet release can be an active yielding of control, demonstrate faith and trust in the practice, and express the meditator's inner awareness of spacious, undistracted clarity. In her instructions for attaining Jhāna, Shaila Catherine emphasizes that Jhāna practice is a training in relinquishment. At every step of the training one is encouraged to let go in more profound and subtle ways. Letting go is both the method and the aim (Catherine 2024, 286).

Arnold, and others, talked about letting go of ego, of suffering, of worrying what people think, and of getting excited about things. “It's letting go of expectations. And overall, when I say letting go of self, I feel like you're letting go of all these notions that society puts on you. You are a child again.” Or, as Calee put it, “Buddha would say let go of desire, aversion and delusion. I suppose that's true, but it's also letting go of thoughts, all those things that are always smashing around in your mind. Just let go.” Metaphors of release can also describe entering the state of Jhāna. Arnold compared the entry to “jumping into a pool.” Shane described it as “sliding into a jacuzzi with the jets turned off.” “Intention is more effective than effort,” explained Jane. “It's not like I have to do anything,” added Arnold. In the realm of Jhāna practice, the act of letting go transcends mere metaphorical significance. It serves as a potent tool, wielding tangible consequences for both the mind and the body.

Sometimes release emerges directly as a response to concentrated effort, something like relaxing after a long run. As Coleen describes, “To enter absorption, our focus has to be diligent and vigilant, otherwise a thought is going to slip in and corrupt it.” Shane explains, “Once the path is cleared, like in the middle of a retreat or after settling in a daily practice, if they're really skilled and their concentration is well established, then the actual entrance into Jhāna can't require any effort.” Note her use of the word “can't.” She is describing diligent effort in preparing the mind, but at some point the assertion of personal effort seems a barrier to entering Jhāna. “It's just the intention and the letting go” she explains, “It requires trust. Dive in, drop in, slide in, or turn away from other things.” Jhāna, it seems, requires both concentration and release. “So, in a way it takes enormous energy. And then in another way you could say it's totally effortless depending on what point in the practice you look at.”

Coleen expands on this connection between concentration and release when she suggests that she begins in the forest, by a tree. “I had a lot going on, and I was just struggling, and I was trying to sit to meditate and I just couldn't. So, I laid down and looked up at this tree, and I was imagining ‘oh, to be a tree and to let go and to be able to just blow with the wind’, and I lay with that sense of asking, ‘what would it be like to just let go?’ And then boom, that's when it happened.” She felt an expansion, loss of self, and utter quietude. Release, in this situation, bred stillness and insight.

She later came to see this moment as her initiation into Jhāna‐like absorption experiences with release at its core. “That's the whole practice. It's all about letting go, and not letting go, and being afraid to let go, and being attached to this life and to my loved ones and to this and that and then letting that go.” Release here reverses her sense of clinging to the world. And, oddly enough, it can be difficult to let go. Holding on may come a bit more easily. “There were a lot of years where I just wasn't going to let go. I just wasn't going there.” Fear kept her from relaxing her guard. But effort is similarly problematic. “Then there were years where I was like, I really want that. I'm going to make that happen. And that doesn't work either.”

I (Brahinsky) asked the obvious counterpoint, “But how does letting go relate to concentration? Because concentration seems like the opposite of letting go.”

“No, I don't think so.”

“Okay, help me.”

“When we think of concentrating,” she explained, “we think that ‘I'm going to take a book and open it and make myself pay attention to this’, right? ‘I'm going to get really focused and concentrate on it’. That experience is different than the concentration that comes with Jhāna. With Jhāna, it starts with ‘I'm going to focus on the breath. I can focus only on this.’” The world around disappears from sensory awareness. But then there is a switch. “When everything else starts to dissipate, that's when you let go.”

Here release is not simply the end of focus, but a precursor to its deepening. Diving into jhānic absorption seems to require a release of effortful focus in a context where attention is not engaging with sensory perceptions or thought. When conditions for Jhāna are ripening, the habit of perceiving oneself as the meditator who is focusing on a predetermined object might fall away, our participants explain. The momentum of attention to the breath or *nimitta* is said to prevent perception from moving toward distractions, as focused attention and trust draw the mind into full absorption. “So, at first, you really concentrate, but then the practice takes on a life of its own, and you can access the sense of opening up that doesn't have that tight quality that normal concentration has. I don't need that tightness anymore. [She holds her hand together tightly. Then they spread back out]. And so, I can let go.” In Coleen's narrative, releasing a tight hold on focus only deepens the absorption.

Similarly, Jory describes how after building concentration, her intention works together with release to enter an absorption state. She prepares by focusing intently. “Once I'm there, I just set this intention. ‘May I enter the first Jhāna, a happiness born of seclusion!’, that's my mind trigger. And then I just let go of everything.” Yet, by the second Jhāna “all the effort goes away. It becomes completely self‐sustaining. You are putting zero effort into it whatsoever.” In our study, practitioners were asked to switch between Jhāna meditation using the breath *nimitta* and mindfulness practice anchored on the physical sensations of breathing, but because entering Jhāna involves release, it sometimes took a strong determination to keep from going there. “The last day of our retreat I had a hard time staying away from Jhāna. The mind just kept wanting to go there, and I had to open my eyes for a few seconds to stop the flow into Jhāna.”

For Jory, letting go of a sense of a personal self who is controlling the experience emerges after the reiterative release of self‐sustaining thoughts. As she put it, “the second that the ‘I’ comes in there, Jhāna just scoots away from you just out of your reach. So, when those self‐affirming thoughts happen, you have to learn to let them go. When you're in Jhāna, they're not there at all. You notice when it arises. It feels like a drain on your energy. It feels disruptive. It feels very unpleasant. And so then you start to notice it when it arises in daily life, and it has that same disruptive feel. So, you start to feel the lack of a solid self. I have started to really perceive that as an experiential knowing, not just conceptual, like, oh, I read in the Buddhist texts that nothing is personal. You start to really internalize that and have an experience of that happening. I credit that to the practice because I don't know that I would have seen that otherwise.”

As the practice evolves, the entrance to Jhāna can feel fully effortless. As Walter explained, “It feels like suddenly you're going from zero to 100 miles an hour without feeling gravity, without feeling acceleration, but you're there. It's like a whoosh and you're in.” And sometimes it feels fully spontaneous, “It just sort of happened, and it happened fast. And I think there was a sense of, like, boom and boom. And then almost like, what is going on?” explained Coleen.

David connected the dots, “Our teacher always says, when you come to a retreat, it's not like going to a conference. You go to a conference, you want to come home with a bunch of good ideas to employ. She said, ‘you're not here to get things. You're here to leave things behind’. As we go through life, we spend a lot of it trying to accumulate all kinds of crap. And really what we should be figuring out is, what don't we need anymore? And it's really a lot of things.” Jhāna practice involves an intentional release of words, concepts and cares, a cognitive practice that has a very visceral effect on experience by enabling a shift into absorption.

## 7 Joy and Arousal During Jhāna Practice

In the Buddhist analysis of cognition described in Abhidhamma texts, the arousing factor of joy (called *pīti* in the Pāli language) is defined as having the specific function of refreshing attention (Bodhi, 85). While Jhāna teachers seem to agree that emotions such as joy or bliss and experiences of pleasure regularly occur during Jhāna practice, they might argue about the details of their techniques, suitability of different meditation objects, required depths of seclusion, and the role that joy, bliss, and the concomitant sense of arousal play in the cultivation of Jhāna. Some teachers include awareness of physical pleasure and sensations in their approach to Jhāna, while other teachers only validate sublime non‐sensual mental pleasure. Some encourage energetic intensity that revs up the bliss, while other teachers require refined delight just sufficient to refresh attention (Catherine 2024, 170). Some encourage meditators to focus directly on the experience of pleasure as their meditation object, while other teachers insist that attention must continue to be directed toward the breath or *nimitta* and consider intense expressions of joy a distraction that must settle prior to the experience of Jhāna. Some teachers apply the term Jhāna to brief experiences that last only seconds or a few minutes, whereas others reserve the term Jhāna for absorption states that could be sustained for long periods such as 30, or even 90 min without being interrupted by other perceptions, thoughts, or sensations (Anālayo 2020; Catherine 2024, 15). But despite these differences, the shared sense that arousal matters inspired us to consider how arousing qualities of joy and pleasure are experienced in the course of Jhāna practice, and the impact that joyful arousal may have on the capacity for sustained focus and the experience of letting go.

When reviewing the descriptions that we gathered from Jhāna practitioners as they reflected on the impact of the states, it was not always possible to carefully parse whether they were talking about the moments before entering Jhāna, during the Jhāna absorption, or after emerging from Jhāna. The method that they followed includes phases in which the mind prepares for Jhāna, approaches readiness, enters into the state, abides deeply immersed in it, emerges from the absorption, reflects on the qualities of the state, and observes the lingering impact on the body and mind. The following descriptions may express highlights of the experience that are not necessarily applicable to every phase of the cycle before, during, and after absorption.

Our participants frequently used imagery that evoked feelings and tangible experiences to convey the quality of their meditation. The meditation method that they practiced, however, demands such a complete absorption with the breath *nimitta* that perceptions of physical sensations, personal thoughts, or changing mental qualities would not be distinguished or analyzed within Jhāna states. Shaila Catherine, a co‐author of this paper and their teacher, describes her pedagogy as one that emphasizes the *nimitta* as the object of focus; changing sensations or fluctuating experiences of joy might be known in preparation phases and after emerging from the absorption, but during deep absorptions little other than the perception of the *nimitta* would be observed. If attention shifted from the *nimitta* to changing mental qualities, feelings, or sensations, this shift might suggest that the mind was not fully absorbed with the *nimitta*. In light states of concentration, according to Catherine, attention may flutter in and out of absorption, diving in for brief dips into Jhāna and then popping out to perceive the qualities of the concentrated mind, and then immersing again in absorption. In the imperfect conditions of our makeshift laboratory or a short retreat, like the one we studied, such light and fluctuating meditative states would be expected.

Additionally, the frequent use of sensory images may simply point to the struggle to put words to spiritual states that are often described as ineffable. One participant, for instance, after comparing Jhāna to sliding into a jacuzzi with the jets turned off, clarified that she doesn't feel sensations of warmth, wetness, or physical pleasure in Jhāna, but that she used the metaphor to convey an experience in which one yields fully into a compellingly pleasant experience.

Jhāna practice seems to produce joy, so much so that teachers sometimes worry about their students smiling too broadly because these facial movements or sensations might draw attention away from the breath *nimitta*. “And our teacher said at one point, stop smiling because you're going to get muscle strain.” Calee then made an effort to contain herself during practice, “So, I try not to smile, but sometimes in the third Jhāna, I know that I'm smiling. Pretty sure.” Calee's metaphors move from ice cream to happy water. “The first Jhāna is kind of like vanilla ice cream. It feels like ‘this is good’. In the third Jhāna the cells of my body, as if they were like water droplets, are happy, and full. There's that sense of, not only is my whole body happy, but there's happiness all around me.”

Sometimes it starts very small. “It was a very simple, small experience, just one morning realizing that concentrating on my breath was very pleasant,” as Kate described it. But then it can grow. “It's very energetic. A lot of energy. There can be kind of a vibration to it at times. I feel infused with energy. I'm compelled to want to straighten up my posture. I'm more awake.”

Likewise, for Jane, at first it is subtle, “the focus on the breath kind of starts to become very pleasant.” But then it grows in intensity. “I wouldn't say it's overwhelming, but it's pretty intense. Happiness and joy. I feel, like in my abdomen, chest, like, in my torso. Happiness. It's just like a warmth, but like butterflies, maybe. It's not calm. It's an exuberant happiness. It makes me smile. Very vibrational. Sometimes I feel it as physical pleasure. Like flushes of intense happiness or joy.”

Sarah describes a tingling throughout the body. “Tingling. Inside, outside, everywhere. It feels like a dissolution of the body. Just like I'm an Alka Seltzer and I've been put in a glass and I'm dissolving. And you feel it head to toe, and bubbling. In fact, the joy gets to be a little bit of a rattle.”

“What do you mean by that?” I asked.

“It's a lot. The joy tends to get out of bounds for me. It's intense. It's very physical. You feel a lot of tingling and this pleasant tensing in the throat or chest. Like you're excited. The best way to describe my sensation of pīti is that sense right before you laugh.”

As David put it, “Jhāna is very euphoric and it's very energetic. It's very blissful. And for me, at least, it had body sensations. There were chills and, you know, like, your hair is standing up on end and it's very pleasurable. So, of course, you don't want to get out of it. You prefer to stay in it. So, you jump into it and get this adrenaline rush, and then you spend the rest of the day trying to replicate that. And of course, you fail. It's not exactly like falling in love, but that's probably the closest ordinary life experience I can think of.”

Walter calls it rapture. “You're just giddy. I mean, it's like, oh, wow. My spine tingles, and sometimes my whole body tingles.” Arnold says, “the hair stands up, or there's a feeling of good vibration going down the spine. I feel it in the back. It's like a physical, subtle vibration throughout the body.”

Perhaps most obviously, joy motivates practice. “It makes it a lot easier to sit for an hour” explained Arnold, “There can be a sense of desire because it's so beautiful. So, there's kind of like a magnetic pull or an attraction.” David understands that joy makes practice easier. “Jhāna is called the wet path, as opposed to Vipassanā (insight meditation) the dry path, because Jhāna creates conditions that make it a little easier to recognize and see things in order to achieve awakening. It greases the skids, if you will.”

To bring things full circle, joy helps with letting go. Walter said he used *pīti* to overcome fear and move forward. He said it was “sort of letting go.” As he explained, joy supported the confidence he needed to release. “The reverse of fear would be just to not worry about it. And that's confidence, faith, whatever you want to call it. But I learned to overcome that by just simply releasing and letting go.” As joy increases, effort decreases to the point where the meditation pulls you in. “It takes effort to get it set up,” Sarah explained. “It doesn't always necessarily pull you, but there's usually some sort of pull.” The pull comes after effort. “The effort comes into letting go and going in. It's that stepping back and letting go to go in.” “The joy is just bubbling up. It's really hard to contain it,” explained Jane.

Practitioners and teachers are quite leery about the appeal of high arousal bliss and joy, which can distract from the spiritual purpose of Jhāna meditation. Jhāna practitioners aim to leverage the energies of pleasure, without becoming attached to them. They describe that as concentration deepens, calmness replaces agitation, and equanimity replaces pleasure, the mind favoring the more subtle qualities.

“In the early practice, when I first started experiencing it, it was quite dramatic. It felt like the energy was unlimited, like touching into a well of joyous energy. The danger, of course, is that the self can attach to the pleasure and excitement. The conceit can attach.” Catherine teaches that the habits of craving, attachment, and conceit are deeply rooted fetters that must be uprooted in the course of meditative development. Her students are asked to notice these mental patterns whenever they arise, in subtle forms such as pride in Jhāna attainment or craving for spiritual bliss, as well as ordinary social manifestations of self‐interest and personal desire. A striking element of the arousing experience of Jhāna meditation, from her perspective, is not that practitioners experience spiritual joy, but that they practice to then let something so beautiful go.

## 8 Release During Speaking in Tongues

Submission and release of control is central to charismatic evangelical worship across multiple registers. Evangelicalism is a form of Christianity built around the idea of being born again after having released the previous sense of self (Bebbington 1989). Charismatics take it a step further with bodily practices that reiterate and enact that sense of letting go. In our ethnographic work, we have encountered practitioners who call speaking in tongues “the Flop” and see the release of the tongue as a means to prostrate before God (Brahinsky 2014). Congregants describe letting go of control of the muscles of their mouth. The nonsemantic phrases that emerge are described as the language of angels, or the voice of the Holy Spirit coming through a person. This release will sometimes expand into leaping, singing and wailing, with the premise that there are few bounds on how people should express themselves when under full submission to God. Yet similar to Jhāna, a tremendous amount of energy also goes into determining which of the more extravagant manifestations of worship are acceptable and which are simply hedonism, or of the devil (Brahinsky 2013, 2020). In this way, charismatic worship links release to a careful process of discernment.

For some practitioners, prayer involves a sense of release around the process of thinking. Prayer allowed Valentin to separate from her thoughts; these thoughts became metaphorical objects that she could pass on—like items in a backpack. “God started taking things out of that backpack,” she explained, “and he was saying, ‘You were never meant to carry this. You are not a failure’. In this way, I was able to experience healing from God, from pain that I had been through as a kid and just the regrets I had for my own actions. I started writing letters to people to ask their forgiveness. It wasn't just my dad. I wanted to have a clean slate to be free to just love on other people, right? I had been too busy carrying my stuff. We all have stuff. But it doesn't incapacitate me like it used to. I used to just replay stuff all the time and have a lot of angst. Life is too short.” Valentin's prayer practice seemed to access her inner thoughts. She packed them up and handed them over to God.

But sometimes charismatic release isquite physical. As Abigail put it, “I used to fall over all the time and I would fight it. I try to stop it because I want to be in control.” When she finally let go—“I was like okay, I give up control”—the experience shifted from a rough metaphorical idea to something deeply physical. “It was powerful. I was just shaking more than I ever have and it was fast and frequent. Like electricity in my body. It felt like fire in my hands.”

And often, charismatic release is powerfully emotional. The most obvious version of this that we saw was when we were asking people to pray in tongues for our research. Skilled practitioners described that, after a matter of seconds, they were able to fully let go of emotional boundaries. We observed them crying straight through prayer blocks of up to 16 minutes until we said it was time to stop.

The basic principle here is that to speak in tongues requires a person to release the tongue. And this bodily submission seems to enable thoughts and emotions to release as well. We are presently wrapping up an initial fMRI study that aims to assess a component of this hypothesis (Brahinsky, Lifshitz, and Luhrmann 2024). Following Newberg et al.'s (2006) initial finding that frontal control mechanisms were deactivated during tongues speech, we predicted that, compared to regular spoken prayer, speaking in tongues would be associated with reduced activation of the supplementary motor area, a brain region implicated in voluntary control of action, the sense of agency, and the routinization of body movement. This would support the idea that speaking in tongues involves a process of letting go of the feeling of control over bodily action. Which is exactly what its practitioners say.

## 9 Arousal During Speaking in Tongues

When asked why she used the word “fire” to describe her sensation, Rhea explained, “That's the word I know to use.” It was not that she literally saw flames. Yet, at the same time, it was also more than a metaphor. Heat was a visceral sensation in her body. Metaphor and body were working in tandem. She quickly moved on to point to other metaphor‐sensory nexus points: “There's been times where it felt like my hands were hot and there's other times where I felt like electricity. Like there was this really weird instance where this guy was like ‘here, put your hand right here [points to her chest] and start praying’. ‘Feel it?’ And I felt energy. It was trippy.”

In one of the earlier quotes, Jane used the image of steam as a metaphor to demonstrate both the fiery nature of the Holy Spirit and its capacity to still her mind and body. The heat dissipating from her body shifted her emotional terrain. “I am just going to go with those words, strength and peace. Coming out, you have the energy, the love, the perspective for the circumstances around you but also to pour out into other people.” As James explained, “it feels like my veins are filled with metal.”

And yet, just as with Jhāna, practitioners of speaking in tongues report moments in the midst of this high arousal that feel like absolute stillness. After a few years of sitting with charismatics just after their highly energized collective worship sessions, we were surprised to hear people talk about periods in the center of that whirlwind that felt quieting, where their minds seemed tranquil. They said it felt like a pond. This did not immediately make sense to us. We heard practitioners describe “utter calm” and the “small quiet voice of God,” probed more deeply, and were told multiple stories of complete stillness, moments in which their mind became so empty and silent that they could hear a whisper from God. Ripples from the smallest rock resonate throughout the pond, they said. For our practitioners, speaking in tongues includes moments of quiet contemplation as well as, and often in the midst of, energized trance.

While fire was accepted by many practitioners as an appropriate metaphor, we also noted a tendency to avoid celebrating these potent emotional experiences as the primary aim of the practice—similar to what we saw with Jhāna. We observed that church leaders make a constant effort to ensure that powerful behaviors and experiences do not supersede the central aspiration of the practice, which in the case of speaking in tongues is to encourage submission and connection to God. Dozens of conversations began with stories of powerful emotional experiences and ended with a reminder that, though the feelings were strong, they were merely the atmosphere experienced in an encounter with something far greater.

## 10 Attention During Speaking in Tongues

The experience of attention during tongues prayer begins with passion. Abigail called it a hunger—a hunger for the word of god. “I really want to dive into the word. I'm just getting more hungry for it. It's crazy. He's given me that hunger.” Valentin talked about “a sense of urgency [in worship], that makes you more alert.” As Candy put it “It's like I want to touch him, so I use my fingers and I rub them as I am feeling this in my heart.” “I don't know, I just want to touch him. I rub my fingers like I am trying to feel him, or I want to see him.”

For Sam, this kind of heat emerges from attention. “All of a sudden, your body's just like ‘It's hot in here’. It's usually when you're pressing into God. Pressing in is inward. It's the simple way of taking my heart and saying, ‘God, no matter how I feel right now, or what things look like right now, I love you and I'm going to worship you’. So that's pressing in, it's an aspect of prayer. It's not about how we feel. It's about knowing who he is which causes us to say, ‘You know what, God, you're worthy and I love you.’” For Sam, ‘pressing in’ seems to be a form of attention perhaps akin to the moment of focus Jhāna practitioners experience just prior to the transition from ordinary consciousness to absorption.

Bright described a sense that attention generates something more than a thought and yet not quite a sensation. I asked, “what gives you the sense that it is God?”

“It's a pure—it's not even a thought. It's just this pure experience. It's more than a feeling, it's like a knowing. It feels, oh here's a word to describe it, peaceful.”

Evangelicals believe that God is always present, always listening and communicating; all they need to do is to pay better attention, and they will find themselves in closer communication with God. This is their rationale for striving for finer‐grained sensory awareness, as they are learning to discern divine messages in the slightest nuances of experience. They describe that through prayer, their senses become increasingly attuned and vivid (for an empirical exploration of this process, see Luhrmann and Morgain 2012). Not only do the sensations gain more meaning, but the bare sensations themselves seem to become more vibrant, clear and detailed. As Amy explained, “I feel like just now that I'm getting closer to him and he is starting to show me things with more clarity.” In other words, what was once especially fuzzy becomes increasingly distinct as a new, finer‐grained set of sensations emerges on the edges of consciousness.

## 11 A Novel Framework: The AAR Spiral

While on the surface, Jhāna and speaking in tongues appear far removed, our phenomenological exploration suggests they share a dynamic interplay between AAR that results in states of absorption. This family resemblance has set us on a research course to incrementally map and compare processes underlying these practices.

Our phenomenological data suggest that in certain contexts and conditions, attention and arousal can coactivate each other, and that this coactivation is supported by a sense of release. As people cultivate the conditions for absorption during Jhāna and while speaking in tongues, they describe feeling increasingly aroused. These heightened states, in turn, seem to amplify their sense of focus. It also seems as if this complementary looping between arousal and attention gains momentum from the experience of release or letting go. And release seems to manifest in several ways. In some cases, release appears to refer to a moment of relaxation that follows a period of high intensity focus and arousal. In other instances, release seems to arise within the throes of focus and arousal, and even to enhance focus. The practitioner reports we describe above suggest not merely an oscillation between arousal, attention, and release but rather a more complex interweaving of these processes, including sometimes even a simultaneous coexistence of the three.

It might seem obvious from our everyday experience that powerful attention can be arousing. Imagine the pleasure or even joy that athletes feel while rock climbing or skiing. Think of someone you love. Yet, we are curious, how does focused attention condition arousal? What is the function of the aroused state in these spiritual endeavors? And what does release have to do with it? We suspect that attention can generate arousal through several mechanisms.

In response, we propose a novel model, the AAR spiral, which builds from several theoretical frameworks to help explain our phenomenological observations. First, we draw inspiration from Fredrickson and Garland's depiction of mindfulness as a spiraling interplay between the experience of decentering from psychological stressors, broadening attention and then reappraising the mindset and situation (Garland et al. 2010, 2015). Put simply, they describe the practice of mindfulness as a metacognitive process that creates psychological distance from our habitual modes of thought and perception and then promotes positive reappraisals that facilitate positive affect and adaptive behavior. This might be analogous in some ways to the dynamic relationship between AAR that we describe here. In our data, it appears that focused attention generates a more aroused mental state which in turn intensifies attention and both enables cognitive and emotional release and is facilitated by this release. We certainly take their model as encouragement to think in spirals.

The interplay we observe between attention, arousal and release during Jhāna meditation and speaking in tongues also makes sense when seen through the lens of predictive coding, which we will explore in more detail below. And we find value also in Lindahl and colleagues' theory of homeostatic neuroplasticity (Lindahl et al. 2014), as we will explain. Finally, this spiraling could seem confusing if we follow the common frame in which arousal and relaxation are posed as opposite ends of a fight or flight process. Instead, the AAR Spiral seems to result in the kind of coactivation of the sympathetic and parasympathetic systems described in Berntson and colleagues' autonomic space model (Berntson, Cacioppo, and Quigley 1993).

## 12 Step One: Attention to Arousal

In both tongues prayer and Jhāna meditation, attention generates arousal. During Jhāna meditation, practitioners employ internally generated objects of focus, such as the *nimitta*. For tongues practitioners, the focus is felt to be external on God, but the practical access to God is through internal sensations (Brahinsky 2012), so they are also looking inward. In both cases, stronger concentration narrows the scope of attention, boosting what computational neuroscientists call the “precision weighting” on the objects of focus (Friston, Parr, and De Vries 2017). The increased attention on an object of interest (e.g., *nimitta*, God) means that the cognitive system is processing more detailed information about the object and thus perceives it more clearly, and has more confidence in its model of the object. Research shows that, in general, uncertainty about perceptions leads to negative affect, while confidence typically causes positive affect (Joffily and Coricelli 2013; Hesp et al. 2021; Seth and Friston 2016). As such, the more we pay attention to an object of experience, the more we may feel confident in our model of that object and therefore feel an influx of positive affect. Our cognitive system then comes to associate the influx of positive valence with that particular perceptual object. This is step one of the spiral: focused attention on an object of perception leads to more clarity and perceptual confidence, which in turn leads to positive valence that the brain associates with that object.

Further support for the idea that focused attention can precipitate arousal comes from our bodies' tendency to compensate for sensory deprivation with sensory overrides–which are experiences of sensory activation without external stimuli. As Lindahl and colleagues note, sensory deprivation during meditation can catalyze sensory experiences such as the emergence of the *nimitta*. When mechanisms that typically filter out extraneous stimuli are reduced, as in sensory deprivation, colors may appear brighter and sensory experiences more intense. Likewise, the serene focus of Jhāna and the repetitive intonations of speaking in tongues may act as “gates” that modulate sensory processing, leading to a state of relative sensory deprivation. By focusing on a singular dimension of experience, such as the breath or God, other sensory input is limited. The quiet of a meditative environment, they suggest, may trigger a homeostatic reaction in the nervous system, which upregulates to compensate for the lack of stimuli (Lindahl et al. 2014). The body may overcompensate by providing access to perceptual experiences (i.e., *nimitta*, God) that become more compelling as they are attended to. These unusually potent perceptions might then support the connection between attention and arousal.

Also, while attention and arousal may construct a positive spiral of mutual reinforcement, focused attention can also trigger a deconstructive process that loosens repetitive thought patterns and frees up the cognitive and emotional resources that were previously invested in those patterns. Neuroscientific research indicates that our brains have limited resources for attention. So, when concentrating our attention on one thing, we may not be able to simultaneously continue to engage in maladaptive thought loops. This understanding is compatible with some Buddhist accounts that suggest that consciousness arises with one object of perception at a time (Ñ. Bhikkhu 1995, 351). When practitioners sustain focus on one object such as breath *nimitta* or God, the momentum of focused attention can increase, and the mind may stop seeking competing perceptions, allowing tranquility and relaxation to pervade this heightened state of energetic focus. His release of energy may be experienced as joy, or arousal. Refreshed and rejuvenated, the mind that emerges from absorption may be more agile (B. Bhikkhu 2000, 87–88) and supportive of novel perspectives, which may underlie what Buddhists call insight or what Christians call knowledge.

## 13 Step Two: Arousal Back to Attention

The second step of the spiral after attention generates arousal is that our attention is naturally drawn to perceptual objects that feel affectively valenced. In other words, affectively‐charged objects are more effortlessly salient, involuntarily drawing our attention toward them (Vuilleumier 2005, Pessoa 2009). Thus, as the object of perception (either God or the breath *nimitta*) becomes more positively valenced through either increased attention and clarity, compensation for sensory deprivation, and/or the release of free energy, then the practitioner's attention becomes more naturally and effortlessly drawn toward the object. This affect‐driven increase in effortless attention then loops back to generate more perceptual clarity about the object, which further increases the positive affect associated with the object, resulting in a mutually reinforcing spiral. This dynamic seems to induce an experience of sustained, aroused attention that can be experienced by the practitioner as increasingly effortless.

## 14 Step 3 (Or Step 0): Release

We suspect that the practice of release plays a pivotal mediating role in the cyclic interplay of attention and arousal in Jhāna and speaking in tongues. The experience and intention to release permeates both practices, particularly in key moments. Participants describe letting go—a release of physical, cognitive and emotional holding and patterns—although Jhāna and tongues practitioners might feel and express release in more or less dramatic ways. Release is especially difficult to define in this context where physical, emotional, and cognitive forms of letting go seem to play back and forth with each other. But the simplest version is that in an atmosphere of increasing ease, words, bodies, and feelings can be released either with clear intention or by virtue of attending elsewhere.

Both practices begin with somatic relaxation: in Jhāna, this involves settling into physical stillness, while in tongues prayer the highly active motion of rapid tongues movement also requires physical relaxation. Both Jhāna and speaking in tongues involve noticing and releasing unwanted thoughts, but each practice follows a distinct emotional and cognitive pathway. In Jhāna practice, individuals actively let go of everyday thoughts and mental patterns, simplifying the emotional landscape and fostering equanimity. While early practice may trigger intense emotions, such as crying or strong sensations, these tend to diminish with experience, according to experienced practitioners. Conversely, speaking in tongues involves letting go of everyday patterns of emotional stability, not simply thoughts, which also simplifies their emotional landscape toward equanimity. They call it “peace.” Cognitive release may invite joyful arousal in preparation for the Jhānas, but as Catherine teaches it, Jhāna practice inclines toward stability and equanimity. For tongues practitioners, the release is often cathartic, with emotional intensity suffusing much of the practice, though they are also cautioned against indulging in pleasure for its own sake. Overall, Jhāna emphasizes cognitive release, while tongues focuses more on emotional release, although the release of emotions seems to then support the release of thoughts, and vice versa. Despite these differences, practitioners of both forms often feel renewed and mentally recharged after emerging from deep absorption, as if something has been literally released from their systems.

Often at the start of practice, but then at key moments throughout, practitioners describe setting an intention for and then actively engaging in a multifaceted surrender—physical, emotional and/or cognitive. When someone has developed a stable focus on the breath *nimitta* and feels ready to go into Jhāna, they intentionally relinquish control to enter the Jhāna states. Similarly, individuals speaking in tongues release control over their speech, often leading to emotional expressivity and cognitive release through which they experience sensory overrides and unusual thoughts. As they feel God's presence intensify, they may choose to lean in and release further. These intentional acts of release create a dynamic interplay with the oscillating patterns of attention and arousal. Each surrender not only deepens the state of absorption but also fuels the cycle, enabling deeper levels of attention, arousal, and further release.

These processes of release may operate in several ways. The spiral of attention and arousal can create an environment of effortlessness and ease, as we have described above. As the object of attention becomes more affectively valenced, maintaining focus becomes easier. Then, as our attentional and emotional systems relax, it becomes easier to let go even further. This sense of ease seems to build upon itself, leading to greater release.

One explanation for this is that sustained attention on a particular stimulus leads to repeated updates and confirmations of the brain's predictive model based on the focused input, reinforcing the brain's model for that stimulus. As a result, a perception of the object of focus may feel more fresh as it is refined and reinforced. Meanwhile, concentration utilizes attentional resources that would otherwise be focused on ruminative thoughts or ordinary sensory inputs. The predictive models for these unattended stimuli are held less tightly which results in less consistency, and perhaps more freedom. As the thought patterns loosen, they may fade from consciousness altogether. The absence of those patterns may engender a feeling of joy or freedom, thus reinforcing the AAR spiral (Laukkonen and Slagter 2021).

Our phenomenological study reveals that release is facilitated by the interplay of attention and arousal that fosters an atmosphere of effortlessness, but is also actively pursued by practitioners through repeated enactments. Practitioners seem comfortable with the paradox of actively surrendering. Following periods of intense focus and arousal, this deliberate yielding—whether to a divine presence or to the process of Jhāna—seems to catalyze relaxation across somatic, emotional, and cognitive spheres. Intriguingly, because this cycle recurs throughout each practice, practitioners report experiencing concurrent relaxation (parasympathetic activation) and arousal (sympathetic activation)—a state not typically emphasized in conventional relaxation research, which instead emphasizes how these systems are usually inversely related. While sympathetic and parasympathetic systems tend to coactivate only in passing, each rising as the other falls, they can indeed operate simultaneously, as suggested by Cacciopo and colleagues' autonomic space model, Berntson, Cacioppo, and Quigley 1993. This sustained arousal in high‐intensity contemplative practices may stem in part from the unusual, prolonged coactivation of both systems, a phenomenon that warrants further exploration and could expand our understanding of autonomic responses in contemplative states.

## 15 AAR As a Conceptual Model

In brief, here is the AAR model as we have developed it so far.

*Step 1*: As we attend to an object of perception, the object becomes more clear, which gives the brain more confidence in its perceptual model of the object, leading to an influx of positive affect and thus encouraging the brain to form an association between the object and positive feelings.

*Step 2*: The emergence of positive affect draws our attention effortlessly to the object and sets in motion a reciprocal cycle between attention and arousal, each dynamically supporting the other.

*Step 3 (which can be step 0 as well)*: Practitioners consciously intend and enact a surrender of control which is facilitated by the emerging experience of effortlessness, cultivated through the spiral of attention and arousal. As this spiral progresses, it supports deeper states of absorption.

## 16 Conclusion

Our venture into the phenomenology of Jhāna meditation and speaking in tongues has led us to observe that, despite their stark differences, these practices both involve a spiraling interplay between attention, arousal and release—a dynamic process we've come to describe as the AAR Spiral. Here we propose a tentative phenomenological model based on our comparative interview data, alongside some preliminary musings regarding underlying mechanisms. We hope that this study demonstrates the value of a comparative approach that examines shared processes across vastly different cultural practices, while also respecting the richness and diversity of these unique contemplative traditions. Pursuing a deeper understanding of the neurocognitive processes underpinning these experiential patterns holds the potential to advance our understanding of the intricate dance between focused attention, arousal, and the art of letting go.

## Author Contributions

All participant names have been anonymized to ensure confidentiality. While Shaila Catherine is a meditation teacher and co‐author of the paper, we also shared drafts of the paper with charismatic evangelicals we work closely with, although they did not have the time to contribute as co‐authors.

## Ethics Statement

All participant names are anonymous, and persons gave their informed consent prior to their inclusion in the study pursuant to UC Berkeley IRB Registration Number: IRB00000455 & IRB00005610.

## Conflicts of Interest

The authors declare no conflicts of interest.

## Data Availability

The data that support the findings of this study are available on request from the corresponding author. The data are not publicly available due to privacy or ethical restrictions.
